# Reversal effect of Jagged1 signaling inhibition on CCl4-induced hepatic fibrosis in rats

**DOI:** 10.18632/oncotarget.18484

**Published:** 2017-06-15

**Authors:** Guiju Tang, Zhihong Weng, Jun Song, Yixiong Chen

**Affiliations:** ^1^ Department of Obstetrics and Gynecology, Union Hospital, Tongji Medical College, Huazhong University of Science and Technology, Wuhan 430022, China; ^2^ Department of Infectious Disease, Union Hospital, Tongji Medical College, Huazhong University of Science and Technology, Wuhan 430022, China; ^3^ Division of Gastroenterology, Union Hospital, Tongji Medical College, Huazhong University of Science and Technology, Wuhan 430022, China

**Keywords:** recombinant adeno-associated virus, Jagged1, hepatic fibrosis, hepatic stellate cells, epithelial-mesenchymal transition, Pathology Section

## Abstract

The role of the Notch ligand Jagged1 in hepatic fibrosis remains to be elucidated. In the current study, we investigated the role of Jagged1 in the activation of hepatic stellate cells (HSCs) and development of hepatic fibrosis in rats. *In vitro*, Jagged1 in HSCs was downregulated and upregulated by Jagged1 siRNA and pcDNA3.1 Jagged1, respectively. The levels of epithelial-mesenchymal transition (EMT) markers and HSC activation markers were assessed using western blot analysis. The proliferation and migration capacity of HSCs were assessed using 5-ethynyl-2′-deoxyuridine (EdU) incorporation and Transwell migration assays. *In vivo*, a recombinant adeno-associated virus type 1 (rAAV1) vector carrying Jagged1 shRNA (rAAV1-Jagged1-shRNA) was constructed and transferred to rat livers *via* the tail vein. Reversion of liver fibrosis and the effect of Jagged1 signaling on EMT were studied using pathological, immunohistochemical and immunofluorescence methods. Our findings revealed that downregulation and upregulation of Jagged1 inhibited and promoted, respectively, HSC activation. The migratory capacity of HSCs was markedly restrained by Jagged1 siRNA. Furthermore, downregulation of Jagged1 suppressed EMT in HSCs. rAAV1-Jagged1-shRNA was generated to treat CCl4-induced hepatic fibrosis in rats. Treatment with rAAV1-Jagged1-shRNA reversed hepatic fibrosis by decreasing EMT. The results of the present study suggest that inhibition of Jagged1 is a potential treatment to ameliorate liver fibrosis.

## INTRODUCTION

Hepatic fibrosis is a wound-healing response to chronic liver injury, which if persistent leads to cirrhosis and liver failure [1]. Although multiple liver cell types play a role in fibrogenesis, hepatic stellate cells (HSCs) have attracted great attention [2]. Fibrosis is reversible, and cirrhosis may regress in some cases [3]. A number of specific antifibrotic therapies have been tried, but the effect has not been satisfactory.

Humans have five Notch ligands comprising Jagged1, Jagged2, Delta-like ligand (Dll) 1, Dll3, and Dll4. Mutations in Jagged1 lead to Alagille syndrome (AGS). It is interesting that patients with AGS do not develop serious fibrotic disease despite the paucity of bile ducts and hence a potentially cholestatic phenotype [4]. Expression of Jagged1 and Dll4 mRNA is seen in normal and diseased liver tissue, whereas expression of Jagged2, Dll1, and Dll3 mRNA is undetectable [5]. Jagged1 mRNA expression is significantly up-regulated in diseased liver tissue. Jagged1 protein has been found in cultured HSCs that develop into myofibroblast-like cells [6].

Our previous study found that blocking the Notch signaling pathway *via* treatment with DAPT, a γ-secretase inhibitor, ameliorates liver fibrosis. The *in vitro* study revealed that DAPT treatment could suppress epithelial-mesenchymal transition(EMT) in a rat HSC line (HSC-T6) [7]. The role of Notch ligands in liver fibrosis remains to be defined. In the present study, we investigated the role of Jagged1, which is a Notch ligand that plays a significant role in the process of liver fibrosis. Jagged1 is activated in rat hepatic fibrosis induced by CCl4, which is a classic drug used to induce liver fibrosis because of its liver toxicity. Furthermore, inhibition of Jagged1 by shRNA delivered *via* recombinant adeno-associated virus 1 (rAAV1) can significantly attenuate liver fibrosis. These results suggest that selective interruption of the Notch ligand Jagged1 may be a novel therapeutic approach for hepatic fibrosis.

## RESULTS

### Gene silencing efficiency of different Jagged1 siRNAs

**Figure 1:**
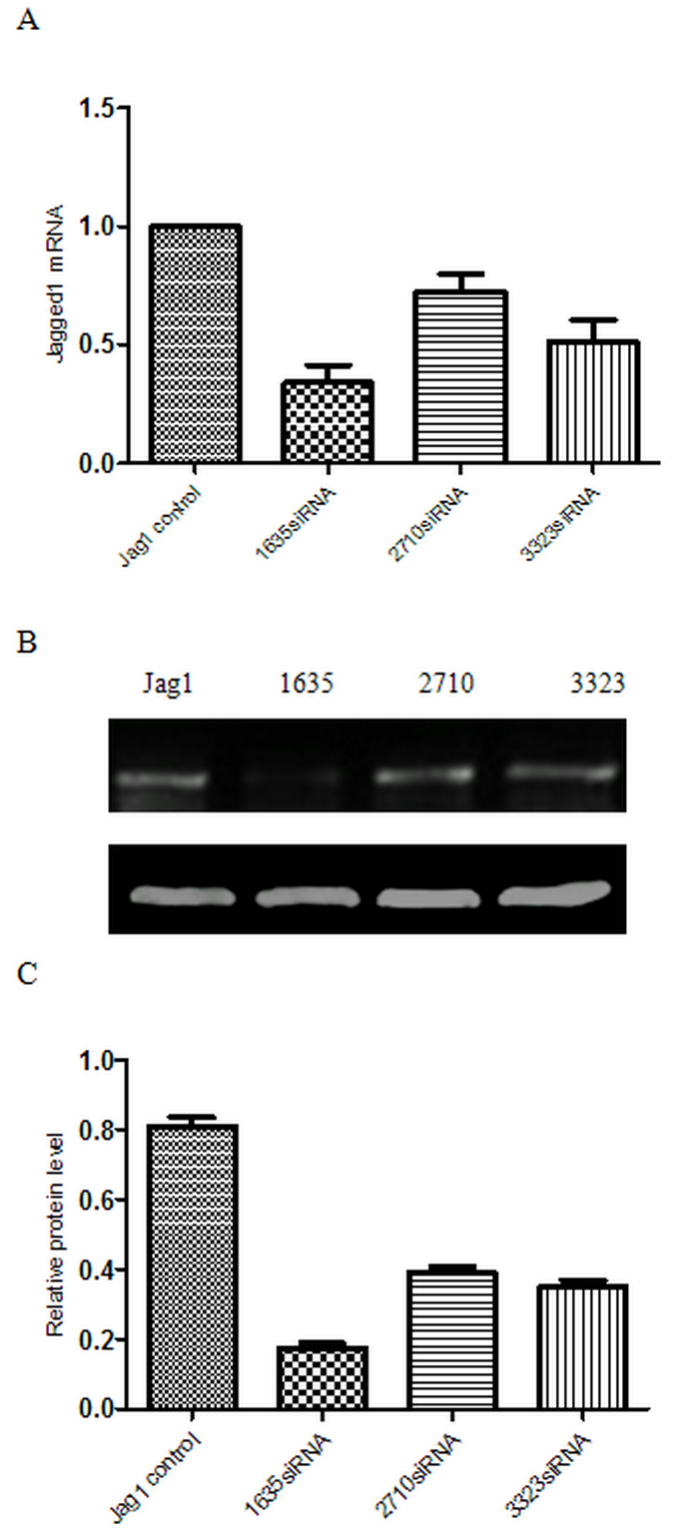
Gene silencing efficiency of different Jagged1 siRNAs Total RNA and protein was obtained from HSC-T6 cells transfected with NC siRNA (NC) and three different Jagged1 siRNAs (1635siRNA, 2710siRNA and 3323siRNA). **A.** The gene expression of Jagged1 was detected *via* real-time RT-PCR 48 h after transfection.The data were quantified *via* comparativeΔΔCT calculation. **B.** Jagged1 protein expression was analyzed *via* western blot 72 h after transfection. **C.** Semiquantitative analysis of the western blot results. The expression was normalized against β-actin. *p < 0.05 *vs* control group.

Three candidate siRNA sequences complementary to various regions of the rat Jagged1 gene were chosen to be tested. These siRNAs were transfected into HSC-T6 cells; Jagged1 mRNA expression was analyzed with real-time PCR 48 h after transfection, and Jagged1 protein levels were analyzed by western blot 72 h after transfection. As shown in Figure 1A, 1635siRNA reduced Jagged1 mRNA expression to 34.1±12.73% at 48 h after transfection, and 2710siRNA and 3323siRNA also suppressed Jagged1 mRNA expression to approximately 72.67±12.34% and 51.57±14.97%, respectively. Furthermore, the silencing efficiency of 1635siRNA at the protein level was confirmed by western blot (Figure 1, B and C).Accordingly, we chose 1635siRNA for use in the subsequent experiments.

### Downregulation and upregulation of Jagged1 affects activation and EMT of HSCs

**Figure 2:**
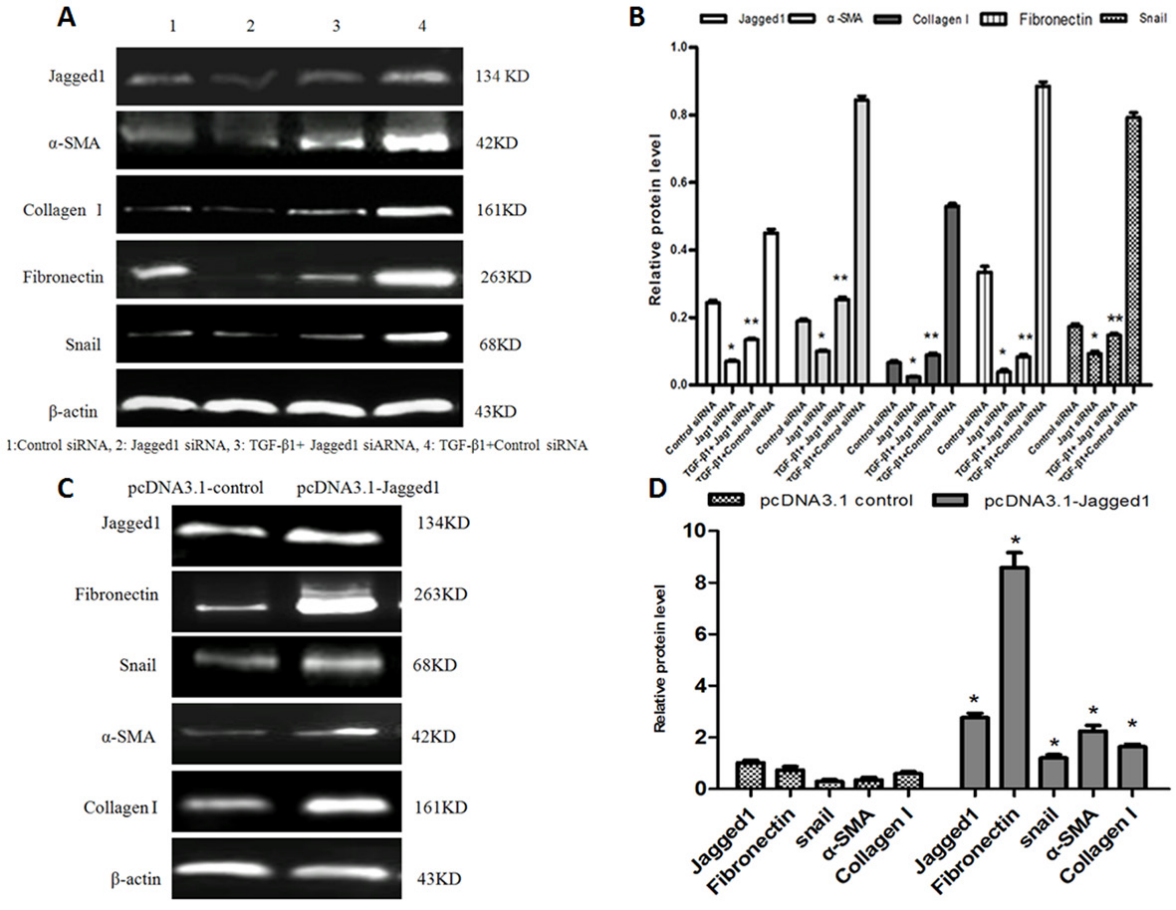
A. Knockdown of Jagged1 inhibited the activation of HSCs and suppressed EMT The expression of Jagged1, α-SMA, collagen I, fibronectin and Snail were detected *via* western blot. **B.** Semiquantitative analysis of the western blot results. The expression was normalized against β-actin. ^*^p < 0.05 *vs* control siRNA group;^**^p < 0.05 *vs* TGF-β1+control siRNA group. **C.** Overexpression of Jagged1 increased the expression of myofibroblastic markers in HSC-T6 cells and promoted EMT. Jagged1, α-SMA, collagen I,fibronectin and Snail were detected *via* western blot. **D.** Semiquantitative analysis of the western blot results. The expression was normalized against β-actin. *p < 0.05 *vs* pcDNA 3.1 control.

To investigate the role of Jagged1 in activation of HSC-T6 cells, 1635siRNA was used to specifically knockdown Jagged1. Western blot analysis showed that knockdown of Jagged1 in HSC-T6 cells suppressed the expression of the myofibroblastic markers α-SMA and collagen I 72 h after siRNA transfection. The expression of Snail and fibronectin in HSC-T6 cells was downregulated (Figure 2A and 2B). However, overexpression of Jagged1 by pcDNA3.1-Jagged1 led to increased expression of α-SMA and collagen I compared with the control cells transfected with empty pcDNA3.1 vector. The expression of Snail and fibronectin in HSC-T6 cells was upregulated (Figure 2C and 2D). Furthermore, TGF-β1 (2 ng/ml) was added to HSC-T6 cells 24 h before transfection with siRNA targeting Jagged1 or control siRNA. Western blot analyses demonstrated that knockdown of Jagged1 antagonized the TGF-β1-induced upregulation effect on HSC activation and EMT in HSCs.(Figure 2A and 2B).

### Downregulation of Jagged1 reduced the migration ability of HSCs but had no significant effect on HSC proliferation

**Figure 3:**
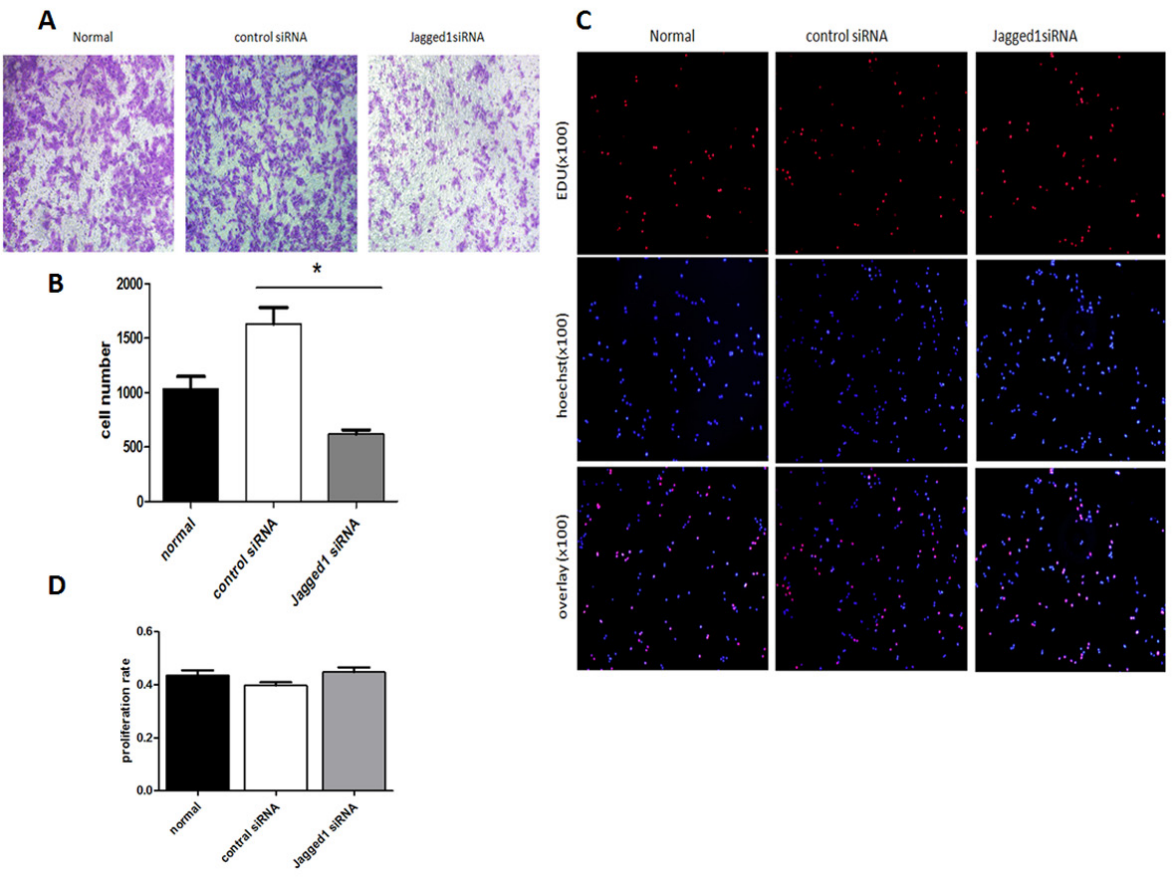
Transwell assays demonstrated that the migratory capacity of HSCs was markedly restrained by Jagged1 siRNA(Figure 3,A and B).*p < 0.05 EdU assays showed that repression of Jagged1had no significant effect on HSC proliferation ( **Figure 3, C** and **D**).

To demonstrate the role of Jagged1 in modulating the biological behavior of HSC-T6 cells, the expression of Jagged1 was downregulated in HSC-T6 cells using Jagged1siRNA, and the resulting transfectants were assessed for cell proliferation and migration ability. Cells transfected with control siRNA served as the control. Transwell assays showed that the migratory capacity of HSCs was markedly restrained by Jagged1siRNA (Figure 3, A and B). However, downregulation of Jagged1 had no significant effect on HSC proliferation. (Figure 3C and 3D)

### Preferential binding of rAAV to fibrotic areas in livers

**Figure 4:**
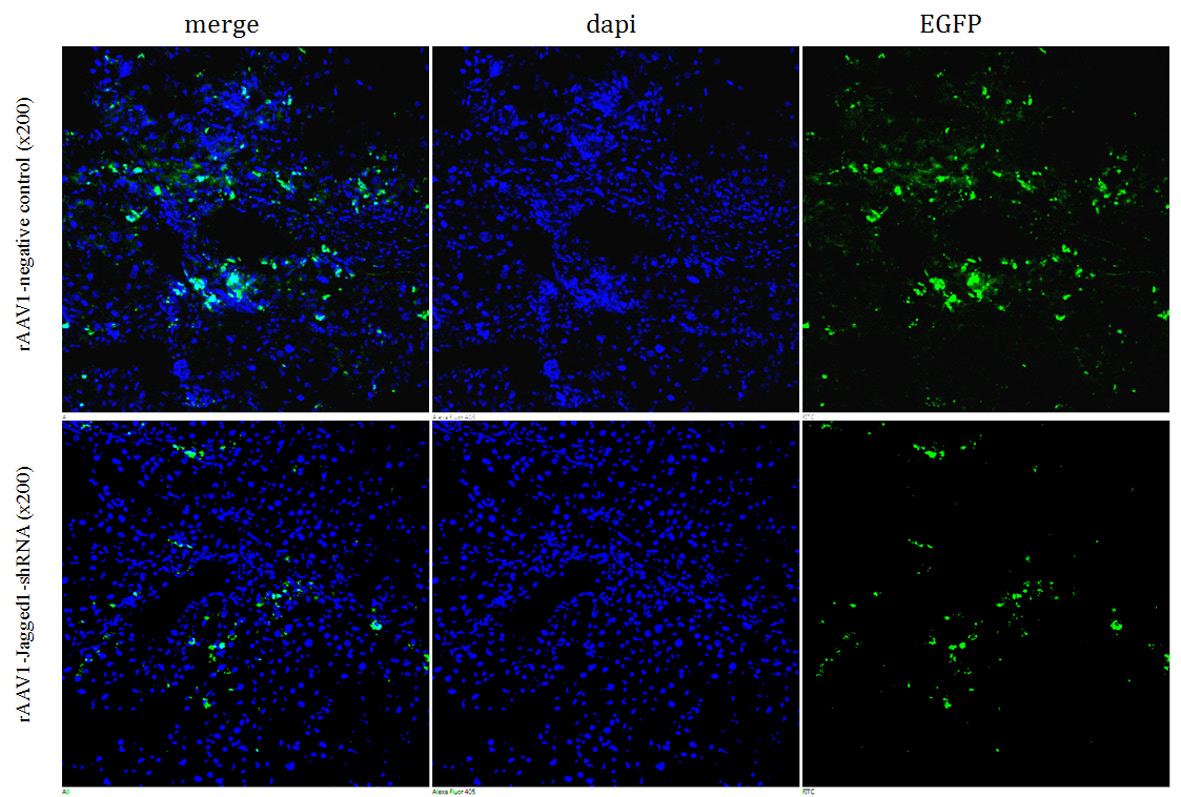
Preferential binding of rAAV to fibrotic areas of livers The expression of EGFP in frozen sections of liver tissue was assessed by confocal laser scanning microscopy. Approximately 30% of cells in the fibrotic liver tissue were observed to express green fluorescence, which was detected predominantly in the fibrotic areas.

To investigate the distribution and infection efficiency of rAAV1-Jagged1- shRNA-EGFP *in vivo*, the expression of EGFP in frozen sections of liver tissue was assessed using confocal laser scanning microscopy four weeks after rAAV1-Jagged1-shRNA-EGFP transfection. Recombinant adeno-associated virusis predominantly expressed in the liver. rAAV1-Jagged1-shRNA-EGFP carries the EGFP gene. After infection, rat liver cells expressed EGFP, which spontaneously emits fluorescence using a 488 nm excitation wavelength. EGFP green fluorescence was detected predominantly in the fibrotic areas of CCl4-induced livers, whereas no expression of green EGFP fluorescence was detected in hepatocytes (Figure 4).

### Inhibition of Jagged1 expression reversed liver fibrosis

**Figure 5:**
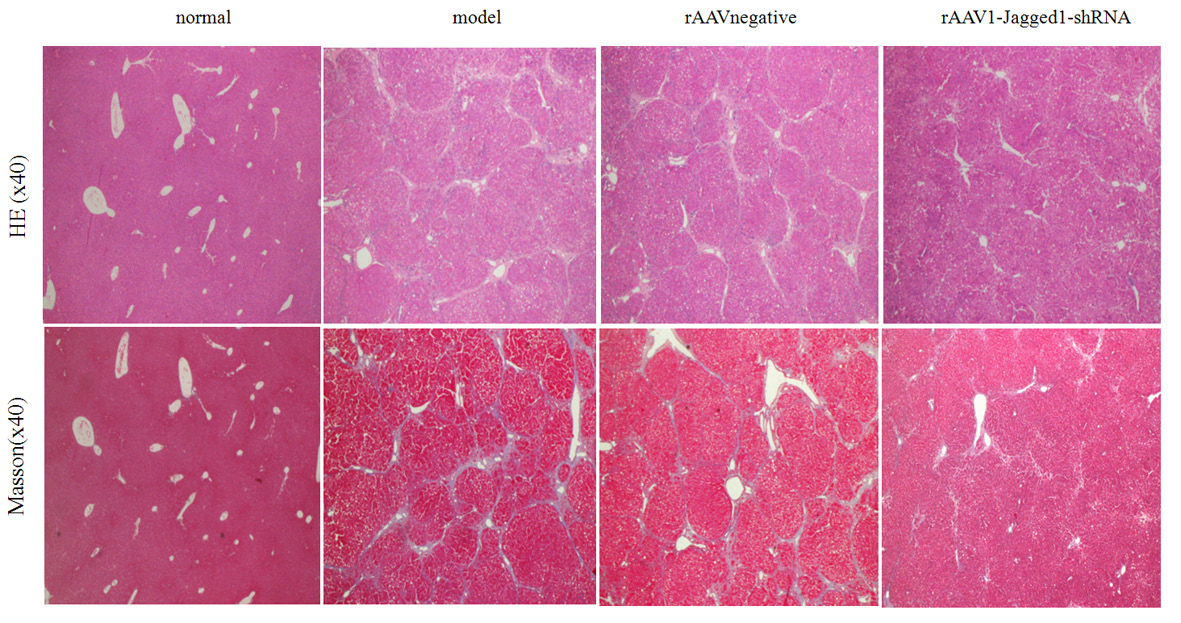
Inhibition of Jagged1 expression reversed liver fibrosis Staining with hematoxylin-eosin and Masson’s trichrome was used to examine pathological alterations and collagen deposition in liver tissues of rats in the normal, model, rAAV1-negative and rAAV1-Jagged1-shRNA groups.

**Figure 6:**
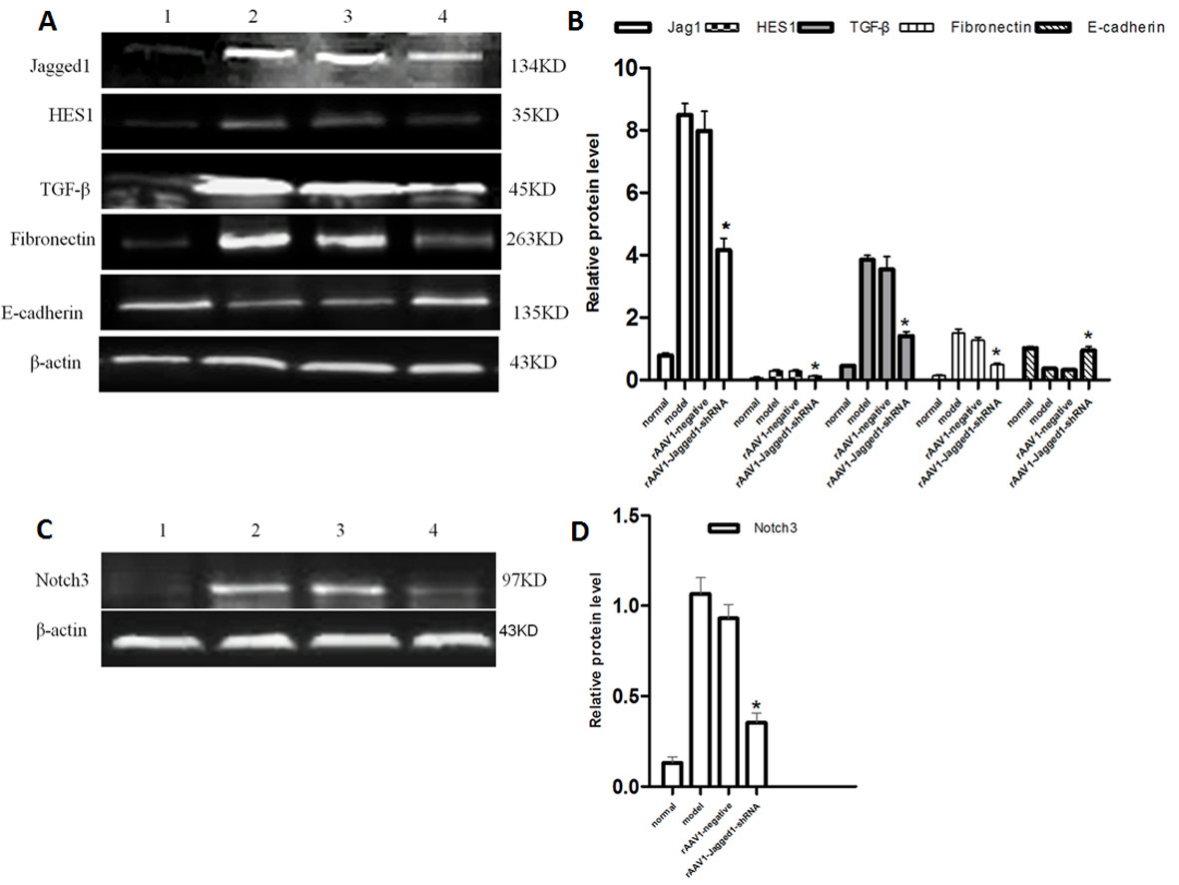
A. rAAV1-Jagged1-shRNA inhibited Jagged1 and ameliorated hepatic fibrosis by inhibiting EMT Western blotting was performed to detect the expression of Jagged1, HES1, TGF-β, fibronectin and E-cadherin in livers from normal, model, rAAV1-negative control and rAAV1-Jagged1-shRNA rats. **B.** Semiquantitative analysis of the western blot results. The expression was normalized against β-actin. *p < 0.05 relative to the model or rAAV1-negative group. **C.** Western blotting was performed to detect the expression of Notch3 in livers from normal, model, rAAV1-negative control and rAAV1-Jagged1-shRNA rats. **D.** Semiquantitative analysis of the western blot results for Notch3. The expression was normalized against β-actin. *p < 0.05 relative to the model or rAAV1-negative group. 1: normal, 2:model, 3:rAAV1-negative control, 4: rAAV1-Jagged1-shRNA.

**Figure 7:**
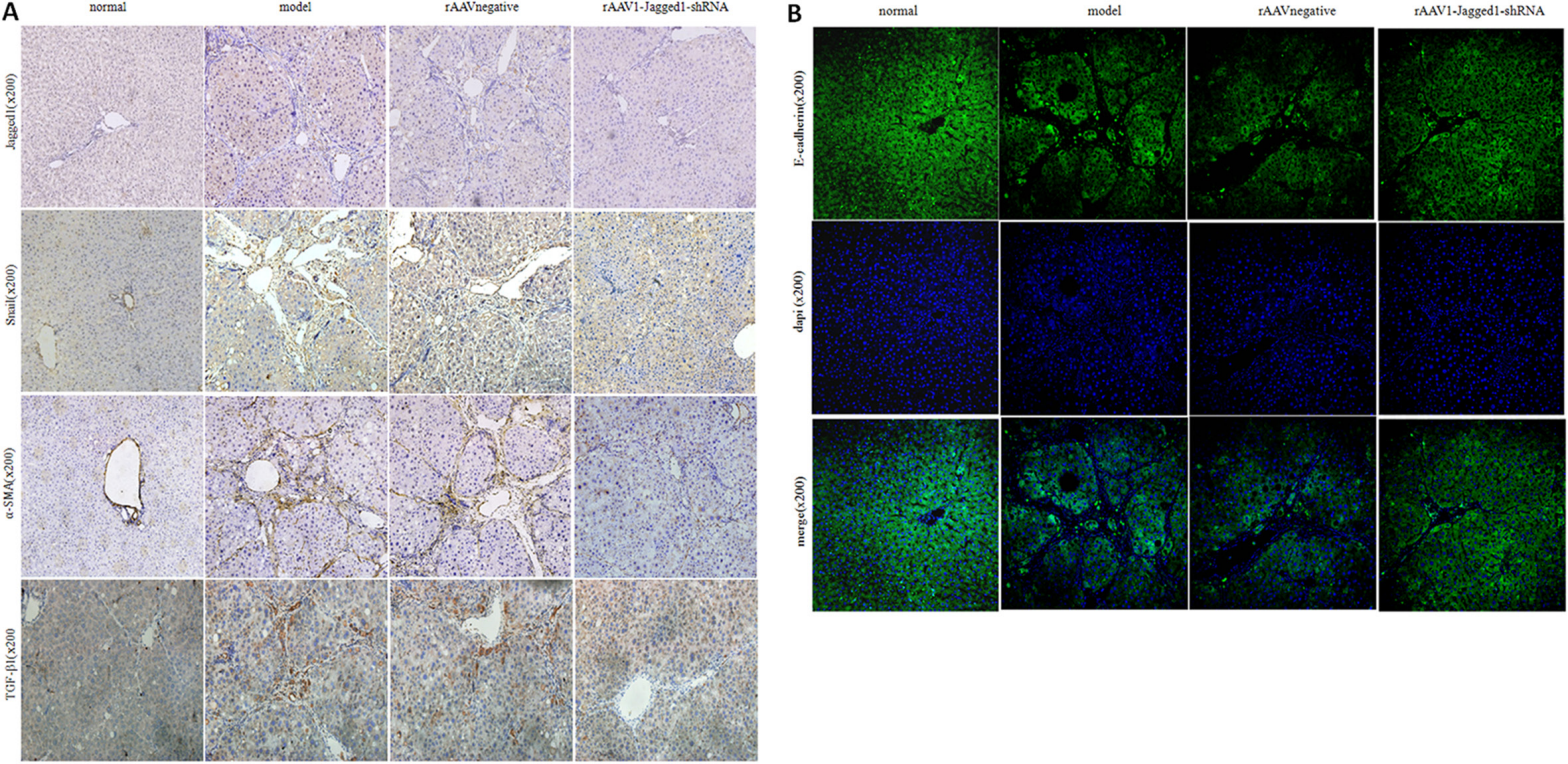
rAAV1-Jagged1-shRNA ameliorated hepatic fibrosis by inhibiting EMT **A.** Immunohistochemical staining was used to detect the expression of Jagged1, α-SMA, Snail and TGF-β1 in livers from normal, model, rAAV1-negative control and rAAV1-Jagged1-shRNA rats. **B.** immunofluorescence were used to detect the expression of E-cadherin (green) in livers from normal, model, rAAV1-negative control and rAAV1-Jagged1-shRNA rats. Nuclei were stained with DAPI (blue).

**Figure 8:**
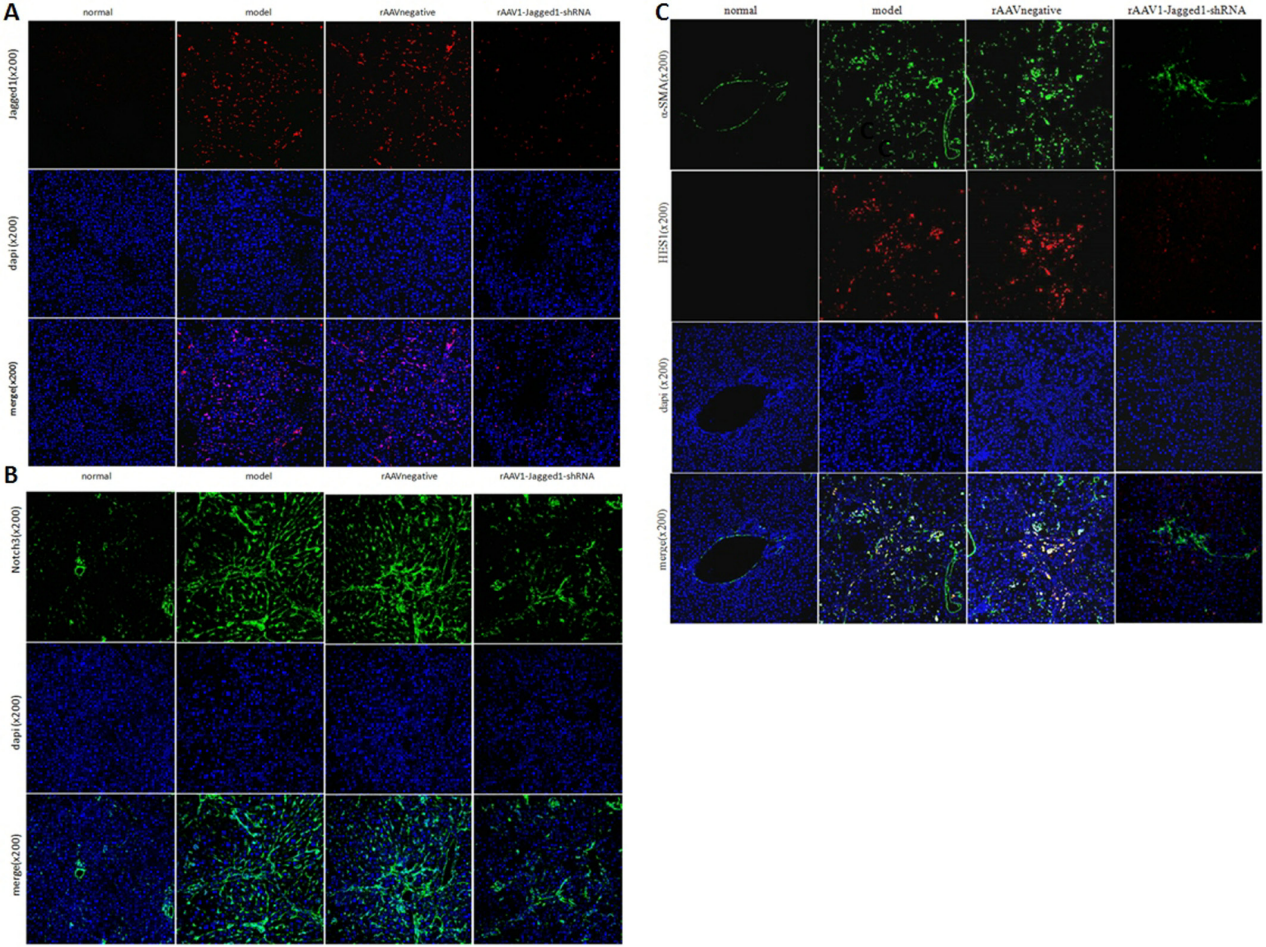
rAAV1-Jagged1-shRNA inhibited Jagged1-Notch3-HES1 in fibrotic livers in rats Immunofluorescence was used to detect the expression of Jagged1(red), Notch3(green), HES1(red) and α-SMA(green) in livers from normal, model, rAAV1-negative control and rAAV1-Jagged1-shRNA rats. Nuclei were stained with DAPI (blue).

Given the potential for inhibition of the Jagged1 signaling pathway in the treatment of hepatic fibrosis, we further evaluated the effects of inhibition of the Jagged1 signaling pathway on hepatic fibrosis *in vivo*. HE and Masson staining were used to investigate pathological alterations and collagen deposition in the liver tissues of rats. As expected, liver fibrosis developed 12 weeks after CCl4 treatment that was characterized by a marked fiber interval formation and significant extracellular matrix (ECM) accumulation, which were secreted by activated HSCs, in the rAAV1-negative control and model group. However, these features of hepatic fibrosis were remarkably attenuated in rAAV1-Jagged1-shRNA-treated rats (Figure 5). The efficiency of rAAV1-Jagged1-shRNA inhibiting Notch signaling was demonstrated by a decreased protein level of Jagged1, Notch3 and HES-1(Figure 6A-6D). As shown in Figure 7A and Figure 8, α-SMA was significantly reduced in the rAAV1-Jagged1-shRNA-treated rats, and this reduction was accompanied by downregulation of Jagged1-Notch3-HES1 signaling.

### rAAV1-Jagged1-shRNA ameliorated hepatic fibrosis by inhibiting EMT

Reversal of liver fibrosis by rAAV1-Jagged1-shRNA was accompanied by an attenuation in the expression of several characteristic proteins of EMT, such as Snail and fibronectin. Through western blot analysis, immunohistochemical staining, and immunofluorescence staining, the expression of Jagged1, Notch3, HES1, α-SMA, TGF-β, Snail and fibronectin was found to be markedly attenuated in rAAV1-Jagged1-shRNA-treated rats compared with rAAV1-negative control-treated rats, whereas E-cadherin expression was increased significantly in rAAV1-Jagged1-shRNA-treated rats (Figures 6-8).

## DISCUSSION

Jagged1 is one of the five cell surface ligands that primarily functions in the highly conserved Notch signaling pathway [8]. Mutations in Jagged1, have been shown to result in AGS. AGS is a multisystem congenital disorder clinically defined by cholestatic liver disease with intrahepatic ductal paucity, structural cardiac defects, ocular anomalies, vertebral body abnormalities, and characteristic facies [9]. Despite the development of ductal paucity, clinical signs of cholestasis often regress as patients approach school age, and there may not be progression to biliary fibrosis or cirrhosis [4, 9].Therefore, further studies are necessary to confirm if intervention of Jagged1 signaling can improve liver fibrosis.

Notch has been reported to be implicated in human fibrotic diseases. Saad et al. reported that the Notch signaling pathway is an important contributor to EMT in renal fibrosis [10].Cao et al. showed that Notch is activated in lung fibrosis induced by bleomycin. Jagged1 upregulation in pulmonary capillary endothelial cells induces Notch signaling in lung fibroblasts, which results in fibrosis [11].Zhu et al. showed that Notch signaling is elevated during PDF-induced peritoneal fibrosis [12]. In addition, the Notch pathway was hyperactivated in skin biopsy samples from patients with scleroderma [13, 14].

In the present study, we showed that Notch signaling was markedly activated in hepatic fibrosis induced by CCl4 in rats. Jagged1-Notch3-HES1 signaling was dramatically upregulated in fibrotic rat livers and was accompanied by overexpression of α-SMA. *In vivo*, we found that overexpression of Jagged1 increased the expression of the myofibroblastic markers α-SMA and collagen Ι in HSC-T6 cells (an HSC line). On the other hand, specific knockdown of Jagged1 with siRNA reduced the expression of those markers and antagonized the activation effect of TGF-β1. Moreover, downregulation of Jagged1 reduced the migration ability of HSCs. These results suggest that the Notch signaling pathway is implicated in hepatic fibrosis. Downregulation of the Jagged1 pathway can inhibit HSC activation.

EMT is a multistep biological process in which epithelial cells change in plasticity by transient de-differentiation into a mesenchymal phenotype [15]. An increasing body of evidence has shown that EMT is implicated in fibrogenesis. Zhu et al observed elevated expression of NICD, Jagged1, and HES-1 during TGF-β1-induced EMT of primary rat mesothelial cells during peritoneal fibrosis [12]. Inhibition of Notch activation by DAPT increases E-cadherin expression but decreases α-SMA and Snail expression in renal fibrosis. Inhibition of Notch signaling by downregulating EMT treats renal fibrosis [10]. Zavadil et al. showed that transforming growth factor-β (TGF-β) induces EMT phenotypes in epithelial cells *in vitro* and *in vivo*. In addition, TGF-β-induced EMT was blocked by RNA-mediated silencing of HEY1 or Jagged1 and by chemical inactivation of Notch [16].

EMT is considered to be a key event in hepatic fibrosis [17-20]. In the present study, we observed elevated expression of fibronectin, TGF-β, and Snail during hepatic fibrosis induced by CCl4 in rats, indicating that EMT participated in this process. Moreover, the expression levels of these proteins were significantly downregulated after rAAV1-Jagged1-shRNA treatment, which decreased Jagged1-Notch3-HES1 signaling. This effect was also demonstrated in cell experiments. Overexpression of Jagged1 increased the expression of the key EMT proteins fibronectin and Snail in HSC-T6 cells. In contrast, specific knockdown of Jagged1 with siRNA reduced the expression of those proteins. Consistent with previous studies [12] [16], TGF-β induces EMT phenotypes in HSCs, but Jagged1 siRNA, blocking the Notch signaling, antagonized the upregulation of EMT induced by TGF-β1 stimulation. Our findings suggest that EMT is involved in the development of liver fibrosis. rAAV1-Jagged1-shRNA treatment can improve hepatic fibrosis through reversion of EMT.

It has been reported that a dramatically increased expression of the rAAV coreceptor, fibroblast growth factor receptor 1α (FGFR-1α), was detected predominantly in fibrotic areas of CCl4-induced livers with cirrhosis compared with normal livers, whereas no expression of FGFR-1α was detected in hepatocytes [21]. These results indicate that rAAV may be an excellent tool for treatment of hepatic fibrosis, specifically interfering with HSCs. Our experimental results showed that the expression of green fluorescence from EGFP was mainly confined to the portal area and fiber spacing, and no obvious expression was observed in liver cells after intravenous injection of rAAV1-Jagged1-shRNA-EGFP in the hepatic fibrosis rat model. In this study, rAAV1-Jagged1-shRNA administration did not affect hepatocyte proliferation (data not shown). Adeno-associated virus (AAV) has shown potential as a gene therapy tool because of its optimal distribution in the liver.

Although further experiments are needed to demonstrate whether other hepatic mesenchymal cells also contribute to hepatic fibrosis through the Jagged1 signaling pathway, the present study suggests that inhibition of the Jagged1 pathway to ameliorate liver fibrosis is a potential treatment, and it warrants further investigation.

## MATERIALS AND METHODS

### Reagents and antibodies

Antibodies against α-smooth muscle actin (α-SMA), Snail, fibronectin, collagen I, E-cadherin, Jagged1, and Notch3 were from Abcam (Cambridge, MA). TGF-β; β-actin and HES-1 antibodies were purchased from Santa Cruz Biotechnology Inc. (USA). Transforming growth factor was purchased from PeproTech (USA). The fluorescent secondary antibodies were purchased from Earthox LLC (USA).

### HSC line and culture conditions

HSC-T6 cells were cultured in Dulbecco’s modified Eagle’s medium (DMEM, Gibco) supplemented with 1% (v/v) penicillin/streptomycin and 10% fetal bovine serum (FBS, Gibco), at 37 °C in 95% air and 5% CO_2_. TGF-β1 (2 ng/mL) was incubated in growth medium for 24 h.

### Transfection of siRNA and plasmid

HSC-T6 cells were seeded into 6-well plates at a density of 1×10^5^ cells/well 24 h before transfection. Then, 5 μL of Lipofectamine 2000 was added into 250 μL of Opti-MEM I for 5 min. Jagged1 siRNA was added to 250 μL of Opti-MEM I for 5 min. They were mixed for another 20 min to form a liposome/siRNA mixture. The mixture was added to the transfection wells, and then, another 1.5 mL of FBS-free DMEM was added to the transfection wells for a total volume of 2 mL, in which the siRNA concentration was 100 nmol/L. The mixture containing siRNA was replaced by DMEM containing 10% FBS after 6 h, and cells were incubated for another 72 h. The following siRNA sequences were used: Jagged1 siRNA, 5’GGCCAAGCCUUGUGUAAAUTT 3’; and the control siRNA sequence, 5’ CAG UAC UUU UGU GUA GUA CAA 3’.

HSC-T6 cells were transfected with pcDNA3.1-JAG1 plasmid and pcDNA3.1-control plasmid using Lipofectamine 2000 following the manufacturer’s instructions. The pcDNA3.1-Jagged1 vector contained full-length Jagged1 cDNA. The siRNA and plasmid were synthesized by Shanghai Genepharma Co. Ltd. (Shanghai, China).

### rAAV1 vectors construct

The shRNAs targeting rat Jagged1 mRNA, the control shRNA, the rAAV1-enhanced green fluorescent protein (EGFP) carrying Jagged1 shRNA (rAAV1-Jagged1-shRNA-EGFP) and rAAV1-NC were designed and produced by Vector Gene Technology Co. Ltd. (Beijing, China).

### Animal experiments

Male Sprague-Dawley (SD) rats weighing 180 to 220 g were obtained from the experimental animal center of Tongji Medical College of Huazhong University of Science and Technology (Wuhan, China). All animal experiments were approved by the Committee on Animal Experimentation of Huazhong University of Science and Technology and performed in compliance with the university’s guidelines for the care and use of laboratory animals. Forty male SD rats were randomly allocated into four groups: normal, model, rAAV1-negative control (rAAV1-NC) and rAAV1-Jagged1-shRNA groups. Rats in the normal group received olive oil (3 mL/kg) twice a week for 12 weeks. In the first stage of the experiment (8 weeks), rats in the model, rAAV1-NC and rAAV1-JAG1-shRNA groups received subcutaneous injections of CCl4 dissolved in olive oil (1:1 ratio) twice a week (3 mL/kg). In the second phase of the experiment (4 weeks), rats in the model, rAAV1-NC and rAAV1-Jagged1-shRNA groups were injected *via* the tail vein with PBS, rAAV1-NC and rAAV1-Jagged1-shRNA, respectively, while the CCl4 administration was in process. The rAAV dose was 5×10^12^ v.g./kg. Rats in all groups were sacrificed at the twelfth weekend.

### Assessment of cell proliferation *via* 5-ethynyl-2’-deoxyuridine (EdU) incorporation

HSCs were seeded in triplicate at a density of 5×10^3^ cells/well in 96-well plates. Cells were transfected with Jagged1 siRNA. After 24 h, cells were fixed with 4% formaldehyde for 20 min. First, the cells were treated with 0.5% Triton X-100 for 10 min for permeabilization. Subsequently, the cells were incubated with EdU stain for 1 h. Finally, the cell nuclei were counterstained with Hoechst33342 for 30 min. The proportion of EdU-incorporated cells were defined as the proliferation rate. EdU Assay Kits were purchased from RiboBio (China). Each assay was performed at least three times.

### Transwell migration assay

Cell migration assays were performed using a 24-well Transwell chamber (Corning, USA). The cell density was adjusted to 8×10^4^ cells with 100 µL of serum-free DMEM in each Transwell insert. The lower chamber was filled with 600µL of DMEM containing 20% FBS. After incubation for 24 h at 37 °C, the upper membrane surface was wiped with a cotton swab to remove non-migrating cells. Migratory cells that traversed to the lower surface of the membrane were fixed with 4% paraformaldehyde for 30 min and stained with crystal violet for counting. Each assay was performed at least three times.

### Histopathology and immunohistochemistry

Liver tissues were harvested and paraffin embedded. The liver sections were stained with hematoxylin-eosin (HE) and Masson’s trichrome. For immunohistochemistry, liver sections were stained with primary antibodies against α-SMA, Jagged1, E-cadherin, Snail and TGF-β1. For immunofluorescence, the secondary antibodies were conjugated with Dylight488 (goat anti-rabbit, green), Dylight488 (goat anti-mouse, green) or Dylight594 (goat anti-rabbit, red). Cell nuclei were counterstained with 4’,6-diamidino-2-phenylindole (DAPI). The staining was visualized and the images were captured using a laser scanning confocal fluorescence microscope (Nikon, Japan).

### Distribution of rAAV1-Jagged1-shRNA after liver infection

Liver samples were snap frozen and stored at -80 °C until further applications. Eight-micrometer-thick frozen sections were used for the observation. The expression of green fluorescence from enhanced green fluorescent protein (EGFP) was visualized, and the images were captured using confocal laser scanning microscopy.

### TaqMan PCR

RNA extraction was performed using TRIzol reagent (Invitrogen, U.S.) according to the manufacturer’s protocol, and RNA was reverse-transcribed using PrimeScript RT Master Mix Kit (Takara, Japan) as described previously. The following TaqMan probe primers were used: Jagged1 primer (forward), GTGGAAGAGGATGATATGGATAAGC and (reverse), CTCCTCTCTGTCTACCAGCGTGTAC; Jagged 1 probe, CCAGCAGAAAGTCCGGTTTGCCA; GAPDH primer (forward), GATGACATCAAGAAGGTGGTGAAG and (reverse), ACCCTGTTGCTGTAGCCATATTC; and GAPDH probe, ACTTCAACAGCAACTCCCACTCTTCCACC.

### Western blot analysis

Liver tissues were homogenized in cold RIPA buffer (Beyotime, China) on ice with a tissue homogenizer, and the lysates were centrifuged at 12,000 rpm for 20 min at 4 °C. Protein was quantified using a BCA assay (Beyotime, China). The protein samples were boiled in protein sample buffer for 10 min, and then, proteins were separated on 10% SDS-PAGE gels and transferred onto polyvinylidene fluoride (PVDF) membranes (Millipore, USA). The membranes were probed with the appropriate antibody for 16 h in 4 °C after being blocked with 10% skim milk in TBST. Horseradish peroxidase-conjugated secondary antibodies were incubated with the membranes at 37 °C for 1 h. Proteinbands were detected by chemiluminescence using BeyoECL Plus (Beyotime, China) with a digital luminescence image analyzer (Biospectrum 600; UVP, USA). The band intensity was assessed using a Gel-Pro analyzer.

### Statistical analysis

All data are presented as the mean±SD of 3 independent experiments. The analyses were performed with SPSS 18.0 software (IBM, US). Differences were considered significant at *p* < 0.05.
